# Quantitative detection of relative expression levels of the whole genome of Southern rice black-streaked dwarf virus and its replication in different hosts

**DOI:** 10.1186/1743-422X-10-136

**Published:** 2013-05-01

**Authors:** Peng He, Jia-Ju Liu, Ming He, Zhen-Chao Wang, Zhuo Chen, Rong Guo, James C Correll, Song Yang, Bao-An Song

**Affiliations:** 1 State Key Laboratory Breeding Base of Green Pesticide and Agricultural Bioengineering, Key Laboratory of Green Pesticide and Agricultural Bioengineering, Ministry of Education, Guizhou University, Guiyang, 550025, People’s Republic of China; 2 National Agricultural Extension Service Centre, Beijing, 100026, People’s Republic of China; 3 Department of Plant Pathology, University of Arkansas, Fayetteville, Arkansas, 72701, USA

**Keywords:** Southern rice black-streaked dwarf virus, Viral gene expression, Real-time quantitative polymerase chain reaction, Rice, Corn, White-backed planthopper, Real-time changes

## Abstract

**Background:**

In recent years, a disease caused by Southern rice black-streaked dwarf virus (SRBSDV) has resulted in significant loss in rice production in Southern China and has spread quickly throughout East and Southeast Asia. This virus is transmitted by an insect vector, white-backed planthopper (WBPH) *Sogatella furcifera* (Hemiptera: Delphacidae), in a persistent propagative manner. Aside from rice, SRBSDV can also infect numerous *Poaceae* plants. However, the molecular mechanism of interaction between SRBSDV and its plant or insect vector remains unclear. In order to address this, we investigated the whole viral genome relative mRNA expression level in distinct hosts and monitored their expression level in real-time in rice plants.

**Methods:**

In this study, a reliable, rapid, and sensitive method for detecting viral gene expression transcripts is reported. A SYBR Green I based real-time polymerase chain reaction (PCR) method was adopted for the quantitative detection of SRBSDV gene expression in different hosts and real-time changes in gene expression in rice.

**Results:**

Compared to the relative mRNA expression level of the whole genome of SRBSDV, P3, P7-1, and P9-2 were dominantly expressed in rice and WBPH. Similarly, these genes also exhibited high expression levels in corn, suggesting that they have more important functions than other viral genes in the interaction between SRBSDV and hosts, and that they could be used as molecular detection target genes of SRBSDV. In contrast, the levels of P6 and P10 were relative low. Western blotting analysis partially was also verified our qPCR results at the level of protein expression. Analysis of the real-time changes in SRBSDV-infected rice plants revealed four distinct temporal expression patterns of the thirteen genes. Moreover, expression levels of P1 and other genes were significantly down-regulated on days 14 and 20, respectively.

**Conclusion:**

SRBSDV genes showed similar expression patterns in distinct hosts (rice, corn, and WBPH), indicating that SRBSDV uses the same infection strategy in plant and insect hosts. P3, P7-1, and P9-2 were the dominantly expressed genes in the three tested hosts. Therefore, they are likely to be genes with the most crucial function and could be used as sensitive molecular detection targets for SRBSDV. Furthermore, real-time changes in SRBSDV genes provided a basis for understanding the mechanism of interaction between SRBSDV and its hosts.

## Background

A disease caused by Southern rice black-streaked dwarf virus (SRBSDV), a notorious member of *Fijivirus*, was first detected in 2001 in Yangjiang City, Guangdong Province, P.R. China and has caused serious loss in rice production in recent years [1-6]. SRBSDV is a plant virus that can infect plants of the Poaceae family, such as rice (*Oryza sativa*) and corn (*Zea mays*) [7-9]. SRBSDV is also an insect virus that affects the white-backed planthopper (WBPH) *Sogatella furcifera* Horváth, the main insect vector species that transmits SRBSDV in a persistent propagative manner [5,7,9,10]. This disease possesses a long latent period and is difficult to detect at its early stages. However, symptoms readily develop it’s the later stages of infection and include dwarfing of plants, stiff leaves, witches’ brooming of leaves, branching at upward rootlets in the nodes, and the development of small waxy swellings on the stems [11,12]. Thus, understanding the interaction between SRBSDV and its hosts is crucial to develop a better understanding of the biology and management of this disease. Xu et al. (2012) constructed two transcriptome groups (viruliferous and non-viruliferous WBPH) and further analyzed potential interaction genes. The genes involved in primary metabolism, ubiquitin-proteasome, cytoskeleton dynamics, and immune responses were up-regulated in viruliferous WBPH [13]. However, the molecular mechanism through which SRBSDV successfully infects and replicates in both plant and insect hosts remains unclear. Thus, whole-gene expression analysis of SRBSDV in the various hosts, including rice, corn, and WBPH is vital for gaining insights into the infection and replication process.

SRBSDV, a double-band RNA virus, is most closely related to but distinct from rice black-streaked dwarf virus (RBSDV), which is also a *Fijivirus* member [11,14]. Comparison of the different genomic segments of SRBSDV with their counterparts in RBSDV suggests that SRBSDV encodes thirteen open reading frames (ORFs) and possesses at least six putative structural proteins (P1, P2, P3, P4, P8, and P10) and five putative nonstructural proteins (P6, P7-1, P7-2, P9-1, and P9-2) [11]. However, the functions of these thirteen genes have rarely been studied. P7-1 induces the formation of tubular structures in heterologous Lepidoptera *Sf9* cells and has been proposed to transport SRBSDV particles [15]. Immunofluorescence staining of P9-1 in *Sf9* cells has shown that P9-1 is sufficient to induce the formation of viroplasm-like structures [16]. The P6 protein has been identified as an RNA silencing suppressor [17]. However, no reports are available to date for other ORFs. The putative function of the translated proteins can only be postulated based on their RBSDV homologs. P1, P2, and P4 are putative RNA-dependent RNA polymerase (RdRp), a core protein, and an outer-shell B-spike protein, respectively [14,18]. P3, P8, and P10 are an inner shell protein, a putative core, and a major outer capsid protein, respectively [14,18,19].

At present, the primary methods for detecting SRBSDV major are reverse transcription-polymerase chain reaction (RT-PCR) [12,20-23] and dot enzyme-linked immunosorbent assay [20,24,25]. However, both methods are only qualitative and not quantitative, and the sensitivity of these assays is limited. SYBR Green I-based real-time quantitative PCR is a reliable, accurate, and effective method that has been extensively applied to detect mRNA levels. Real-time PCR has been recently used in the detection of plant viruses, such as *Epstein*–*Barr virus*[26], *Pseudorabies virus*[27], *cauliflower mosaic virus*[28], *Tenuivirus*[29], *rice tungro bacilliform virus*, and *rice tungro spherical virus*[30]. Recently, two papers have reported on the quantitative PCR (qPCR) detection of SRBSDV based on a single gene. Zhang et al. (2013) used the P4 gene as a detection gene to conduct qPCR for the simultaneous detection and differentiation of RBSDV and SRBSDV [31]. SRBSDV titer in rice plants was determined using qPCR based on P10 [32]. However, simultaneous detection of the real-time mRNA expression level of the whole genes in hosts has not been conducted so far. In this study, a qPCR method was adopted to simultaneously analyze the whole-gene expression levels of SRBSDV in distinct hosts (rice, corn, and WBPH) and monitor changes in their expression in real-time in rice. The results of this study are crucial for understanding the interaction between SRBSDV and its hosts. And further to provide molecular information for investigating the function of viral genes. Moreover, this study may serve as a basis for future investigations on interactions between SRBSDV and its major plant hosts and insect vector.

## Results

### Analysis of the relative mRNA expression level of SRBSDV genes in rice, corn, and WBPH

**Figure 1:**
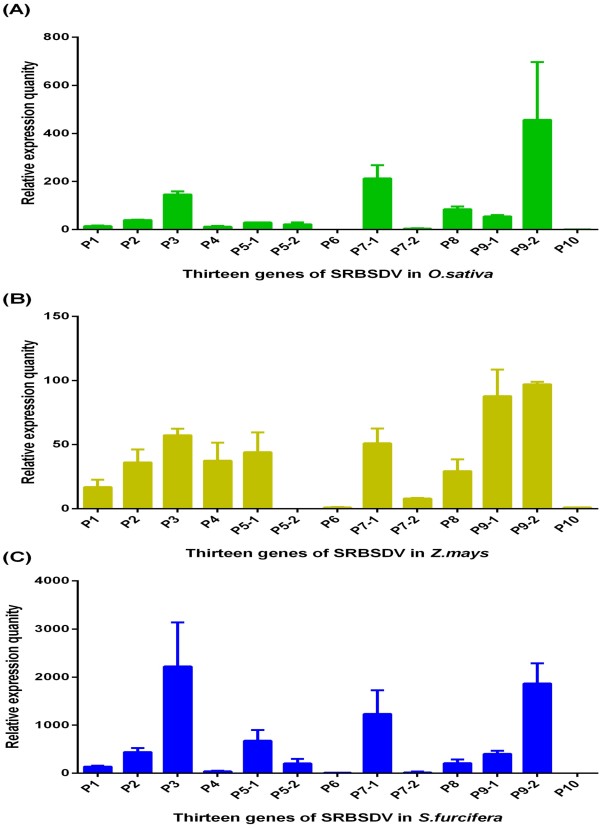
Relative mRNA expression levels of SRBSDV in (A) rice (green bars), (B) corn (earth yellow bars) and (C) WBPH (blue bars).

The goal of our study was to provide basic information on interactions between SRBSDV and its distinct hosts. A qPCR method was constructed to investigate the relative mRNA expression level of SRBSDV genes in infected rice, corn, and WBPH. The relative mRNA expression levels of all thirteen SRBSDV genes were quantified according to the 2^-ΔΔCt^ algorithm (Figure 1 and Table 1). The relative mRNA expression level of the whole SRBSDV genome displayed similar patterns in rice, corn and WBPH. For instance, P3, P7-1 and P9-2 showed relatively high expression in all three hosts. In rice plants, the expression levels of P7-1, P9-2, and P3 are 456.50-, 212.42- and 146.29- higher, respectively, than that of P10. In corn plants, the expression levels of P9-2, P3 and P7-1 were 97.06-, 57.38- and 50.96-fold higher, respectively, than that of P10. In WBPH, the expression levels of P3, P9-2, and P7-1 were 1567.84-, 1142.97-, and 862.69-fold higher, respectively, than that of P10. Furthermore, the level of P6 expression was relative low in all three hosts. In rice and corn plants, P6 expression was only approximately 31% and 0.07%, respectively, of the P10 expression level.

**Figure 2:**
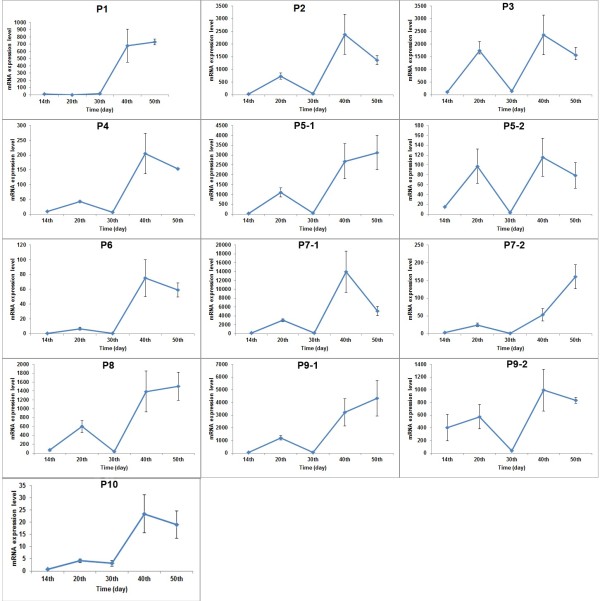
**Temporal mRNA expression levels of 13 SRBSDV genes in *O. sativa.***

**Figure 3:**
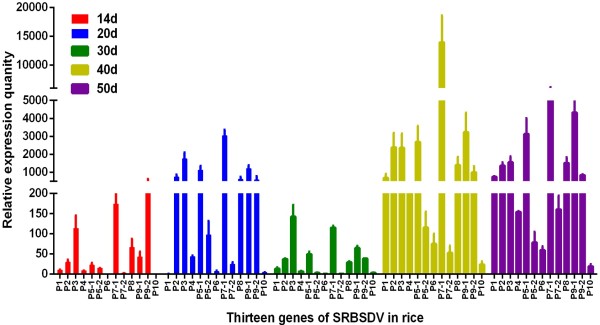
**Temporal mRNA expression levels of 13 SRBSDV genes in *O. sativa.*** The red, blue, green, yellow, purple, and dark red bars indicate of *O. sativa* at 14, 20, 30, 40, 50 d.a.i, respectively.

**Table 1:** Relative mRNA expression levels of SRBSDV genes in distinct hosts

**Gene name**	***Zea mays***	***Oryza sativa***	***Sogatella furcifera***
**P1**	16.82 ± 2.91	14.19 ± 2.09	78.66 ± 42.51
**P2**	36.10 ± 5.12	39.57 ± 1.16	262.18 ± 126.16
**P3**	57.38 ± 2.56	146.29 ± 8.76	1567.84 ± 461.50
**P4**	37.41 ± 7.05	12.42 ± 2.26	27.48 ± 9.74
**P5-1**	44.08 ± 7.77	29.50 ± 0.40	449.93 ± 159.57
**P5-2**	0.07 ± 0.001	22.19 ± 5.35	148.89 ± 40.06
**P6**	0.97 ± 0.19	0.31 ± 0.001	6.70 ± 3.36
**P7-1**	50.96 ± 5.79	212.42 ± 39.16	862.69 ± 261.07
**P7-2**	7.92 ± 0.28	3.84 ± 1.08	17.57 ± 0.57
**P8**	29.35 ± 4.59	84.72 ± 7.74	143.36 ± 47.37
**P9-1**	87.81 ± 10.34	54.93 ± 3.82	234.45 ± 118.27
**P9-2**	97.06 ± 0.97	456.50 ± 170.05	1142.97 ± 512.12
**P10**	1.00	1.00	1.00

**Table 2:** Temporal expression pattern of SRBSDV genes in rice

**Gene name**	**Day 14**	**Day 20**	**Day 30**	**Day 40**	**Day 50**
**P1**	9.96 ± 0.90	0.28 ± 0.07	13.37 ± 3.56	678.35 ± 226.12	730.52 ± 40.98
**P2**	29.41 ± 6.02	731.21 ± 122.02	37.28 ± 1.10	2380.62 ± 793.54	1363.34 ± 174.79
**P3**	113.28 ± 32.10	1737.93 ± 360.04	142.54 ± 29.14	2363.30 ± 787.77	1553.93 ± 316.65
**P4**	8.57 ± 0.46	43.05 ± 3.19	6.52 ± 1.11	205.49 ± 68.50	153.88 ± 2.26
**P5-1**	22.37 ± 5.44	1109.14 ± 234.76	49.51 ± 5.57	2681.72 ± 893.91	3123.61 ± 871.97
**P5-2**	14.85 ± 0.16	97.21 ± 34.72	3.16 ± 0.92	115.72 ± 38.57	78.50 ± 26.34
**P6**	0.23 ± 0.05	6.71 ± 1.76	0.47 ± 0.06	75.12 ± 25.04	59.23 ± 9.48
**P7-1**	173.71 ± 66.53	3033.53 ± 325.33	115.40 ± 4.52	13955.59 ± 4651.86	5081.56 ± 1048.93
**P7-2**	2.51 ± 0.15	24.18 ± 5.14	0.75 ± 0.07	52.80 ± 17.60	160.33 ± 33.94
**P8**	66.54 ± 20.60	597.94 ± 132.96	29.40 ± 2.45	1387.03 ± 462.34	1508.68 ± 316.38
**P9-1**	42.72 ± 12.46	1200.19 ± 179.52	64.78 ± 5.39	3233.23 ± 1077.74	4330.48 ± 1391.08
**P9-2**	403.25 ± 207.70	574.79 ± 194.67	38.59 ± 0.38	993.75 ± 331.25	831.74 ± 45.99
**P10**	1	4.17 ± 0.79	3.13 ± 1.12	23.44 ± 7.81	19.05 ± 5.70

### Temporal expression of SRBSDV genes in rice plants

To investigate the temporal expression of SRBSDV genes in rice plants and further lay the foundation for understanding the SRBSDV infection process, an qPCR method was adopted to detect the relative mRNA expression levels in rice plants at 14, 20, 30, 40, and 50 days after infection (d.a.i.) (Figures 2 and 3; Table 2). All gene expression levels were relative low on day 14 and rapidly increased from day 14 to day 20 (except those for P1). However, by day 30, all gene expression levels decreased (except for P1), and all genes (except those of P1) continued to increase on day 40, and then finally remained relatively stable on day 50. The thirteen genes of the SRBSDV genome showed four different expression patterns in the periods of day 14 to day 50. In the first pattern, certain genes (P2, P4, P5-1, P6, P7-1, P7-2, P8, P9-1 and P10) showed rapidly increased in expression levels with two rapid increases between 14 and 20 d.a.i and between 30 and 40 d.a.i. In the second pattern, the expression level of P1 sharply declined from 14 to 20 d.a.i. On day 20, the expression level of P1 was only approximately 30% of that on day 14. Additionally, from day 30 to day 40, its expression level rapidly increased by approximately 50-fold. In the third pattern, P9-2 displayed a relatively stable expression level in all detected time points, showing a less than 2-fold variation between days 20 and 40. In the fourth pattern, for genes P3 and P5-2, the expression levels rapidly increased from day 14 to day 20, decreased to day 30, and then finally recovered on day 40. In summary, P3, P7-1 and P9-1 displayed more consistent higher expression levels at all time periods sampled, P9-2 showed the highest expression levels, but did not have higher expression levels than the other genes at the remaining sample time points. Conversely, P1 displayed lower expression levels in the early stages of infection and relatively higher expression levels at later sample points.

### Western blotting verification

**Figure 4:**
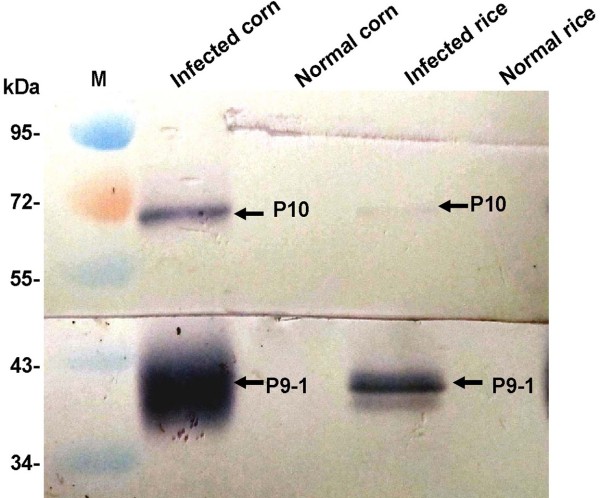
The western blot results of SRBSDV-infected rice and corn plants.

mRNA levels cannot be used as surrogates for corresponding protein levels without verification. To confirm the result of RT-qPCR, we carried out western blot assays using polyclonal antibodies against P10 and P9-1 on the rice and corn samples (Figure 4). The P9-1 protein was more abundant than P10 both in infected corn and rice, which was consistent with our qPCR data.

## Discussion

It has been previously reported that SRBSDV is a dsRNA virus whose genome lacks poly-A [7,11]. In this study, Oligo-(dT)18 primers were used as anchor primers to simultaneously synthesize the single-stranded cDNA templates of the whole thirteen genes of SRBSDV. We have identified that in SRBSDV-infected *Z*. *may*, poly-A is added to P10 and P7 mRNA (Additional file 1). We propose that in the processing of primary RNA transcripts to produce mature mRNA molecules, the viral mRNA uses poly-A polymerase of the host to add poly-A to increase the stability of the molecule and avoid degradation by the ribozyme in host cells. Further research should clarify whether poly-A generally exists in all SRBSDV genes and investigate the molecular mechanism by which this occurs.

In distinct hosts (rice, corn, and WBPH), the thirteen SRBSDV genes displayed some similarities in expression levels suggesting that SRBSDV uses the same strategy to infect and replicate in these hosts. P3, P7-1 and P9-2 were the dominantly expressed genes in rice and WBPH. These genes also displayed relatively high expression levels in corn, suggesting that they have important roles during infection of hosts by SRBSDV. The P7-1 protein in SRBSDV is a virus movement protein that induces the formation of tubular structures in insect cells [15]. The observed high expression of P7-1 suggests that the formation of tubular structures is important for SRBSDV infection after the host cell is invaded. P3 and P9-2 are thought to encode an inner shell protein and a non-structure protein, respectively [11]. However, the molecular functions of these two genes have not yet been characterized in detail. The observed high expressions of P3 and P9-2 suggest that they have important roles in SRBSDV infection. Further studies should verify the significance of their functions. The P6 gene, a suppressor of SRBSDV, was found to be expressed at a relatively low level. The NS3 protein, a suppressor of the rice stripe virus, displayed the highest expression levels in the rice plant and insect vector [29]. The high expression level of P6 may indicate that it is sufficient for suppression of immune responses in plant and insect hosts. Notably, several interesting differences can be considered. Expression levels of most genes relative to P10 were higher in rice and WBPH than in corn. Thus, corn may exhibit stronger immune response against SRBSDV infection. Although P9-1 displayed high expression level in corn, but it showed low expression level in rice and WBPH. P9-1 has been characterized to be involved in viroplasm formation and viral replication inside the infected cell [16]. The high expression level of P9-1 may indicate that SRBSDV hastens its replication against the strong immune response of corn. Furthermore, most genes showed higher abundance in WBPH than in rice and corn. This finding may be attributed to more intensive replication and assembly of SRBSDV in insect hosts than in plant hosts. These high-expression genes could be used as target genes because of their greater sensitivity than other genes (P10 is a common gene for SRBSDV detection) in disease diagnostics and molecular biology studies of SRBSDV. To verify this speculation, a RT-PCR method was adopted for the detection of single SRBSDV-infected WBPH using P9-2 and P10 primers. SRBSDV-infected WBPH was successfully detected by using the P9-2 primers. However, no detection was evident in two parallel tests when using the P10 primers (Additional file 2).

A qPCR method was used to trace the expression levels of the SRBSDV genes in SRBSDV-infected rice on days 14, 20, 30, 40 and 50. On day 20, only P1 (putative RdRp) displayed a down-regulation among the 13 genes. On day 30, expression levels of the other 12 genes were reduced to 5.10, 8.20, 15.15, 4.46, 3.25, 6.93, 3.80, 3.09, 4.92, 5.40, 6.71 and 75.16% of day 20, respectively. Reduction rate of the 12 genes was approximately 90%, except for P10, which had a reduction rate of approximately 24%, close to that of P1 on day 20. The increase rate of P1 on day 30 was 46.75%, which was very close to the average increase rate (56.02%) of the other 12 genes. Additionally, except those of P1, The expression levels of the other 12 proteins decreased on 30 days. It was proposed that the mechanism of changing of virus amount at plant host was deemed to relate with plant immunity [33]. In many plant virus, like as Alfalfa mosaic virus (AMV), Cucumber mosaic virus (CMV), Tomato spot wilt virus (TSWV), Tobacco mosaic virus (TMV) and so on, the concentration of virus will reach at high peak at 7–10 days after infecting plant host, then virus concentration will be decreased at a lower levels [34]. At present, many proteins, genes, materials and signal pathways were found to possess the activity of inhibition replication of virus under the stress of virus infection, for instance, pathogenesis-related proteins, N gene, phytoalexin and SA signal pathway [35]. Moreover, P5-1 and P5-2, P7-1 and P7-2, as well as P9-1 and P9-2 are in the same segment of SRBSDV dsRNA. However, significant differences in expression levels were found even between ORFs in the same segment. In addition, expression level of P9-1 was higher than that of P9-2, except on day 14. P9-2 was suggested to be more important during the early stages of SRBSDV infection because of its dominate expression. Future studies should investigate the function of these genes and the factors leading to difference in their expression levels.

## Conclusion

These results provide a basis for further studies on the infection mechanism and interaction rules between SRBSDV and its hosts. A SYBR Green I-based real-time qPCR method was used to simultaneously analyze the expression levels of 13 SRBSDV genes in distinct hosts (rice, corn, and WBPH) and to monitor real-time changes in their expression levels in rice. P7-1, P9-2, and P3 were the most dominantly expressed genes in the three hosts, suggesting that they have significant roles in the SRBSDV infection process and that they could be targets for SRBSDV diagnosis. Moreover, similar expression patterns of SRBSDV were observed in distinct hosts, which suggested that the same strategy was taken by SRBSDV to infect different hosts. Real-time changes in SRBSDV gene expression levels in rice were analyzed at different time points after infection. Four different expression patterns were found in 13 genes and the time point corresponding to P1 down-regulation was also identified.

## Methods

### Virus, plants, and insect vector resources

SRBSDV-infected rice plants collected from Shidian County, Yunnan Province, PR China were confirmed by RT-PCR [20]. WBPHs collected from Shidian County were fed with SRBSDV-infected rice plants in an artificial climatic chamber at 26 ± 1°C with a light/dark condition of 16/8.

### Transmission and detection methods of SRBSDV

Second-generation WBPH nymphs of third to fourth WBPHs propagated on SRBSDV-infected rice plants were collected and starved for 2 h. The insects were then transferred to virus-free rice (Keyou 21) or corn (Zhengdan 518) seedlings at two- to three-leaf stage in a test tube. The insects were allowed to feed on the seedlings for 24 h. The plants inoculated with confirmed viruliferous WBPH were subjected to RT-PCR detection after 10 d. Total RNA of WBPH, rice, and corn was extracted using RNAiso Plus (TaKaRa) following the manufacturer’s protocol. RT-PCR was conducted using One Step RT-PCR Kit Ver. 2 (TaKaRa) with primers S10F/S10R and P9-2-F/P9-2-R. The cycling parameters were as follows: 1 cycle at 50°C for 30 min; 1 cycle at 94°C for 2 min; and 40 cycles of 94°C for 15 s (denaturation), 58°C for 30 s (annealing), and 72°C for 45 s (extension). RNA (5 μL) was added in each RT-PCR system. mRNA level of the *18 s rRNA* gene was used as positive control. The primers are listed in Additional file 3.

### Quantitative real-time PCR

After 10 d of SRBSDV infection, the mRNA expression levels of rice leaf, corn leaf (at least three replications with six individuals each), and WBPH (three replications with 20 individuals each) were analyzed in distinct hosts. For analysis of SRBSDV multiplication in rice, additional samples in the same group from infected rice were collected on 20, 30, 40, and 50 d.a.i. Total RNA was extracted using an RNAiso Plus Kit (TaKaRa, Dalian, China) following the manufacturer’s instructions. The RNA level was detected using ultraviolet spectrophotometry and agarose gel electrophoresis. Single-stranded cDNA templates were synthesized using 2 μg of total RNA from various samples with Oligo-(dT) 18 primer as anchor primers (Additional file 1). Moloney murine leukemia virus reverse transcriptase (TaKaRa, Dalian, P. R. China) was used for cDNA synthesis. The reaction was conducted at 42°C for 1 h and terminated by heating at 70°C for 15 min. The qPCR reaction for each sample was performed on an iCycle iQ (Bio-Rad, CA, USA) using 20 ng of cDNA template and 10 μM gene-specific primers designed using Beacon Designer 7.7 (PREMIER Biosoft International, CA, USA). The primers used are listed in Additional file 3. The reactions were run for 30 s at 95°C, followed by 40 cycles of 95°C for 5 s and 60°C for 30 s. The mRNA levels were measured via qPCR using SYBR Premix Ex Taq™ (TaKaRa, Dalian, P. R. China). Afterwards, fluorescence was measured via melting curve analysis at 55°C to 95°C to detect a single gene-specific peak and verify the absence of primer dimer peaks. Under these conditions, a single and discrete peak was detected for all primers tested. Non-template reactions (replacing cDNA with free RNase H_2_O) were used as negative controls. A 10-fold dilution series was used to construct a relative standard curve for determining PCR efficiencies and further quantification analysis. All primers exhibited 90% to 100% amplification efficiencies in all experiments. Each reaction was run in triplicate. The mRNA level was quantified in relation to expression of rice *18S rRNA* (GenBank Acc. No. AK059783). Primer pair for the 13 SRBSDV genes was designed to amplify a 70 bp to 250 bp product (rice and corn *18S rRNA* primer as described before [29]; WBPH *18S rRNA* primer as described before [13]), which was verified by nucleotide sequencing and agarose gel electrophoresis (Additional file 4). The means and standard errors of the histograms were obtained from averages of three independent replicates. Relative copy numbers of SRBSDV genes were calculated using the 2^−ΔΔCt^ method [36], and the P10 gene was set as unity.

### Western blot

Western blot assays were conducted using P9-1 and S10 polyclonal antibodies as described previously [20,24]. The protein supernatant of rice and corn were collected and separated by 12% SDS-PAGE electrophoresis using the same protein amount. Separated proteins in the gels were transferred electrophoretically onto a PVDF membrane at 90 mA for 1.5 h. The blotted membrane was cut off at about 50 kDa and two membrane pieces were blocked with 5% skim milk in TBST buffer. After washing the membrane three times with TBST for 15 min, the membranes were incubated with P9-1 and P10 polyclonal antibodies at 5 μg mL^-1^. The bound antibodies were detected by horseradish peroxidase reaction at 1:3000 dilutions.

## Competing interest

The authors declare no conflicts of interest related to this manuscript.

## Authors’ contributions

BAS, SY, and PH designed the study. BAS, PH, and JJL wrote the paper. ZCW, JJL, PH, MH, ZC and RG collected the samples. PH and JJL performed the qPCR tests. PH, JJL, and MH analyzed the data. BAS, SY, JC and PH thoroughly revised the manuscript. All authors read and approved the final manuscript.

## Supplementary Material

Additional file 1 **A JPEG file named, “The 3’RACE results of P7 and P10 in *Z.mays.”*** The SRBSDV sequences of “p7” and “p10” used for these alignments have the following Genbank numbers: JQ034354 and JQ034357, respectively. 7–1, 7–4, 7–5, 7–7, 10–4, 10–5, 10–6 and 10–7 were the sequencing results. Red shadows indicate the polyA region. 3’ RACE was conducted using the BD SMART™ RACE cDNA Amplification Kit (Clontech). The 3’ gene specific primers (GSP) for P7 and P10 were P7-3GSP: 5’-TTGGCAAGCGATGGAAAGAAGATGG-3’ and P10-3GSP: 5’-GCCAACAATTTATTGAAGGCGGATCG-3’. Click here for file

Additional file 2 **A JPEG file named, “Detection of SRBSDV using P10 and P9-2 in *S.furcifera.”*** To validate the sensitivity of P10 and P9-2 primers for detecting SRBSDV infection in *S*. *furcifera*, two SRBSDV-infected *S*. *furcifera* plants were selected. PCR products were analyzed via electrophoresis using 3% (w/v) agarose gel. Electrophoresis was performed using an electrophoresis meter (DYCP-31BN, Liuyi instrument factory, Beijing). The reaction mixture was placed in a 200 μL centrifuge tube. The reactions were conducted at 94°C for 30 s, followed by 40 cycles of 94°C for 30 s, 63°C for 30 s, and 72°C for 1 min. Click here for file

Additional file 3 A word file named, “Primers used for the detection and expression level analysis of SRBSDV.” Click here for file

Additional file 4 **A JPEG file named, “Agarose gel electrophoresis and RT-PCR analysis of SRBSDV genes in distinct hosts.”** These methods were used to detect the thirteen SRBSDV genes in three distinct hosts, namely, *O*. *sativa*, *Z*. *mays and S*. *furcifera*. Preliminary tests were performed with each primer by RT-PCR. PCR products were analyzed via electrophoresis using 3% (w/v) agarose gel. Electrophoresis was performed using an electrophoresis meter (DYCP-31BN, Liuyi instrument factory, Beijing). RT-PCR reactions were performed using C1000 (Bio-Rad, CA, USA) and 10 μM gene-specific primers designed by Beacon Designer 7.7 (PREMIER Biosoft International, CA, USA). The reaction mixture was placed in a 200 μL centrifuge tube. The reactions were conducted at 94°C for 30 s, followed by 40 cycles of 94°C for 30 s, 63°C for 30 s, and 72°C for 1 min. The 13 putative gene fragments were present in all three SRBSDV hosts, as indicated by the presence of target bands. Click here for file
