# Supplementary figures and images for: Quantitative detection of relative expression levels of the whole genome of Southern rice black-streaked dwarf virus and its replication in different hosts
Source: Virol J. 2013 May 1;10:136. doi: 10.1186/1743-422X-10-136 (PMC3655032; doi:10.1186/1743-422X-10-136)

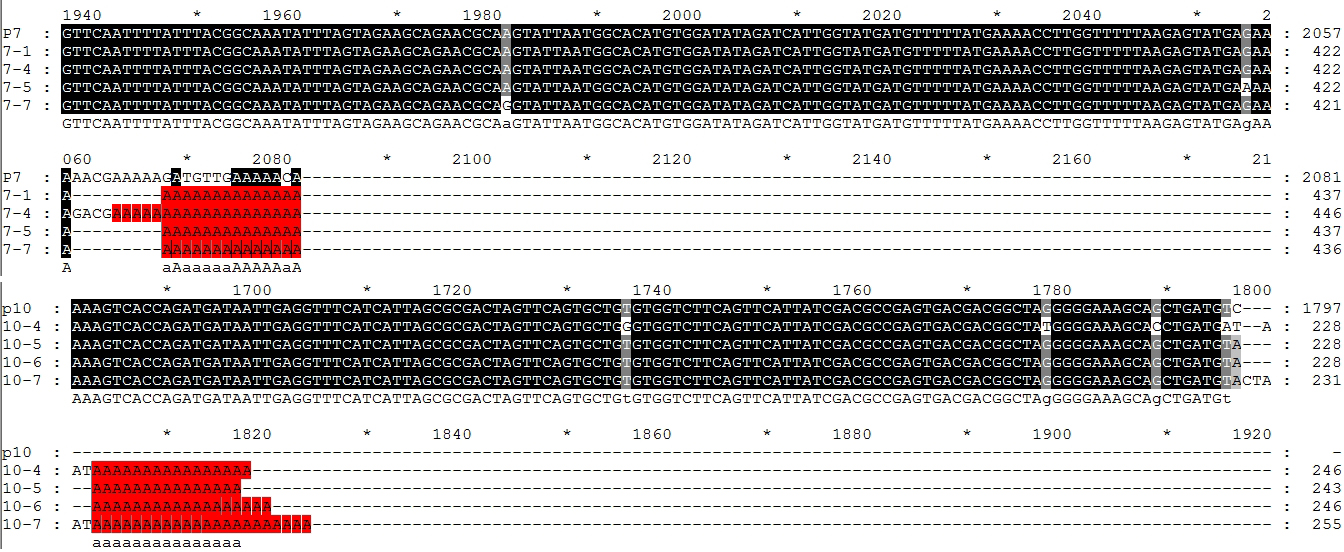

Supplement: Additional file 1 — A JPEG file named, “The 3’RACE results of P7 and P10 in Z.mays.” The SRBSDV sequences of “p7” and “p10” used for these alignments have the following Genbank numbers: JQ034354 and JQ034357, respectively. 7–1, 7–4, 7–5, 7–7, 10–4, 10–5, 10–6 and 10–7 were the sequencing results. Red shadows indicate the polyA region. 3’ RACE was conducted using the BD SMART™ RACE cDNA Amplification Kit (Clontech). The 3’ gene specific primers (GSP) for P7 and P10 were P7-3GSP: 5’-TTGGCAAGCGATGGAAAGAAGATGG-3’ and P10-3GSP: 5’-GCCAACAATTTATTGAAGGCGGATCG-3’. [file 1743-422X-10-136-S1.jpeg]

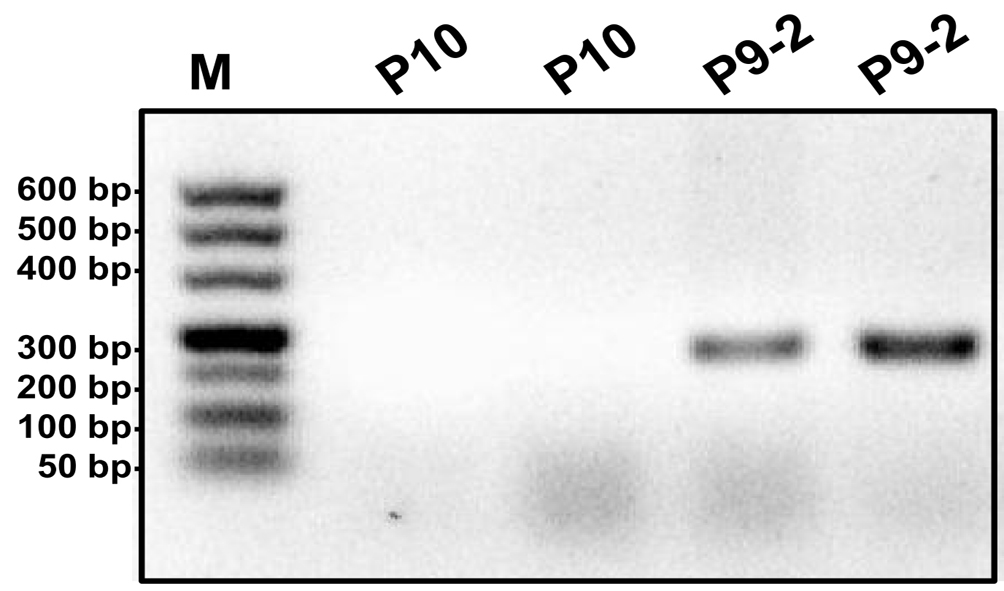

Supplement: Additional file 2 — A JPEG file named, “Detection of SRBSDV using P10 and P9-2 in S.furcifera.” To validate the sensitivity of P10 and P9-2 primers for detecting SRBSDV infection in S. furcifera, two SRBSDV-infected S. furcifera plants were selected. PCR products were analyzed via electrophoresis using 3% (w/v) agarose gel. Electrophoresis was performed using an electrophoresis meter (DYCP-31BN, Liuyi instrument factory, Beijing). The reaction mixture was placed in a 200 μL centrifuge tube. The reactions were conducted at 94°C for 30 s, followed by 40 cycles of 94°C for 30 s, 63°C for 30 s, and 72°C for 1 min. [file 1743-422X-10-136-S2.jpeg]

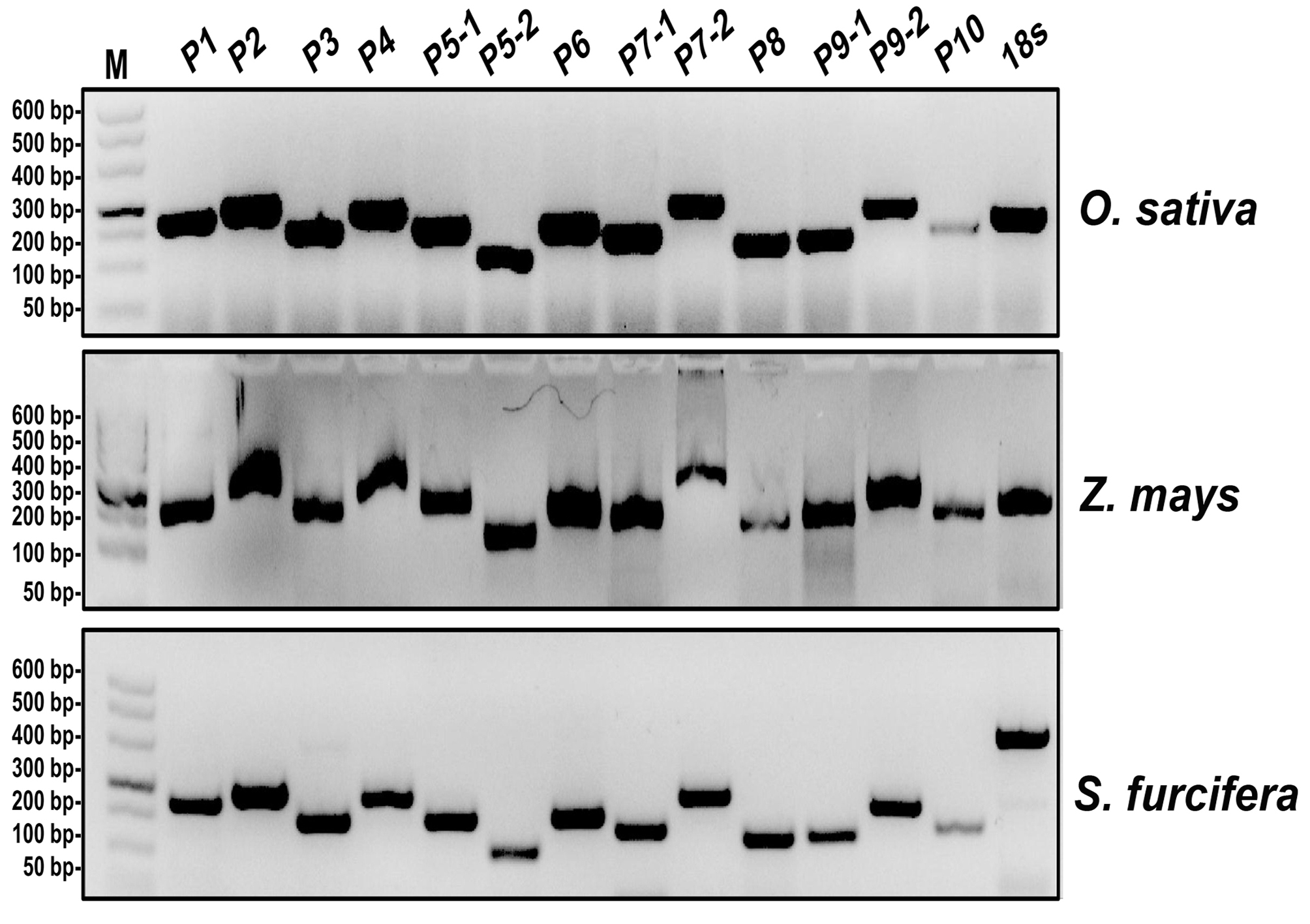

Supplement: Additional file 4 — A JPEG file named, “Agarose gel electrophoresis and RT-PCR analysis of SRBSDV genes in distinct hosts.” These methods were used to detect the thirteen SRBSDV genes in three distinct hosts, namely, O. sativa, Z. mays and S. furcifera. Preliminary tests were performed with each primer by RT-PCR. PCR products were analyzed via electrophoresis using 3% (w/v) agarose gel. Electrophoresis was performed using an electrophoresis meter (DYCP-31BN, Liuyi instrument factory, Beijing). RT-PCR reactions were performed using C1000 (Bio-Rad, CA, USA) and 10 μM gene-specific primers designed by Beacon Designer 7.7 (PREMIER Biosoft International, CA, USA). The reaction mixture was placed in a 200 μL centrifuge tube. The reactions were conducted at 94°C for 30 s, followed by 40 cycles of 94°C for 30 s, 63°C for 30 s, and 72°C for 1 min. The 13 putative gene fragments were present in all three SRBSDV hosts, as indicated by the presence of target bands. [file 1743-422X-10-136-S4.jpeg]
