# Supplementary material for: Quantitative detection of relative expression levels of the whole genome of Southern rice black-streaked dwarf virus and its replication in different hosts
Source: Virol J. 2013 May 1;10:136. doi: 10.1186/1743-422X-10-136 (PMC3655032; doi:10.1186/1743-422X-10-136)
Supplement: Additional file 3 — A word file named, “Primers used for the detection and expression level analysis of SRBSDV.” [file 1743-422X-10-136-S3.docx]

**Additional file 3 Primers used for the detection and expression level analysis of SRBSDV**

| Primer name | Sequence (5′→3′) | Amplicon length (bp) | Target gene  (putative function) |
| --- | --- | --- | --- |
| P1-F | GACGCTCAGTTCAATATCATT | 173 | RdRp |
| P1-R | ATCGCAGCATCTACATCA |  |  |
| P2-F | CAAACTCATCACGACCAGAAGAAA | 195 | Core protein |
| P2-R | CAAATGTTGTGGTATAGGAGGGAAC |  |  |
| P3-F | ATTATGTTGAAGCGGATGGTTG | 151 | Structural protein  Inner shell Protein |
| P3-R | GCGATATGTTACTGGAATGGTGAG |  |  |
| P4-F | ACACAAGCCAGAAGGAAT | 184 | Structural protein B-SPIKE |
| P4-R | CTCATAATCACGACAACCAAT |  |  |
| P5-1-F | GTCATTCACTCGTCATTCG | 152 | Structural protein |
| P5-1-R | ATTCCATCCAACTCTGCTA |  |  |
| P5-2-F | CGATGTCTGACTTATCTTCTAC | 106 | Non-structural protein |
| P5-2-R | AGAAACTTTCATATCACGACAA |  |  |
| P6-F | GAACCTGACAATACTGAACAT | 155 | Gene silencing suppressor |
| P6-R | ATCAACAGCATACGCAATAG |  |  |
| P7-1-F | AAACGAAATACAAGAAATGAGAAT | 136 | Tubular protein |
| P7-1-R | ACTACAACTAACGGAACTGA |  |  |
| P7-2-F | TAAGTCAAGTATGTTACCAATGTT | 192 | Non-structural protein |
| P7-2-R | TGCGTTCTGCTTCTACTAA |  | Nuclear localization |
| P8-F | CCTCATTCGCTGGCATAT | 123 | Core structure |
| P8-R | GTGTAACATCTGATTCGCAAT |  |  |
| P9-1-F | AACGACCAACCAACAAGA | 128 | Viroplasm |
| P9-1-R | GTTCCATCAATGAGGTAGTTC |  |  |
| P9-2-F | AATCCTTGCTGTATATCATTCTT | 176 | Non-structural protein |
| P9-2-R | TACCTCCATTGAACACTTGT |  |  |
| P10-F | ACGAACTAACTGGACTGT | 138 | Capsid protein |
| P10-R | TCTTACGCAACGATGAAC |  |  |
| Rice-18S rRNA-F | ATGGTGGTGACGGGTGAC | 159 | Rice-18s rRNA |
| Rice-18S rRNA-R | CAGACACTAAAGCGCCCGGTA |  |  |
| WBPH-18S rRNA-F | ACAAGTATCAATTGGAGGGCAAGTCTGG | 250 | WBPH-18s rRNA |
| WBPH-18S rRNA-R | ATGCACACAGTATACAGGCGTGACAAG |  |  |
| S10F | CTCCGCTGACGGTTTAGAAG | 242 | Capsid protein |
| S10R | GGTCGTAACCGCCATAGTGT |  |  |
